# Monostotic Fibrous Dysplasia of the Rib: A Case Report

**DOI:** 10.1155/2012/690914

**Published:** 2012-12-10

**Authors:** Asha Mahadevappa, Sapna Patel, Sunila Ravishankar, Gubbanna V. Manjunath

**Affiliations:** Department of Pathology, JSS Medical College, JSS University, No. 1036, 5th Main 10th Cross, 1st Stage Vijayanagar, Karnataka, Mysore 570017, India

## Abstract

Fibrous dysplasia is a noninherited bone disease in which abnormal differentiation of osteoblasts leads to replacement of normal marrow and cancellous bone by immature bone with fibrous stroma. Monostotic fibrous dysplasia accounts for 28% in the ribs. It is often asymptomatic and incidentally detected on radiographs. As with many bone abnormalities, it can be superimposed by the formation of aneurysmal bone cysts. We report a case of a 70-year-old lady who presented with swelling on the chest wall of 20-ear duration and sudden increase in size for 8 months. Radiologically, X-ray and CT scan showed an expansible lesion of the medullary cavity with a ground-glass centre and thinning of cortex of the 5th rib. The resected lesion was a firm, well-defined solid, grey-white expansile mass replacing the medullary cavity. Histopathologically, benign fibrous spindle areas with disorganized irregular bony trabeculae were seen. Hemorrhagic spaces lined by osteoclast-like multinucleated giant cells were also noted. The diagnosis was fibrous dysplasia with aneurysmal bone cyst changes. Although fibrous dysplasia with aneurysmal bone cyst is rare, it should be taken into account in differential diagnosis of the rapidly growing solitary rib lesion.

## 1. Introduction

Fibrous dysplasia (FD) is a noninherited developmental bone disorder in which abnormal differentiation of osteoblasts lead to replacement of normal marrow and cancellous bone by immature woven bone with fibrous stroma [1–3]. It can be monostotic (single bone) or polyostotic (multiple bones). Any bone may be affected, the long bone, skull, and ribs most often [2, 4–6]. FD of the ribs accounts up to 30% of all benign chest wall tumors and monostotic forms are about four–six times more common than polyostotic forms [2, 4, 7]. It is usually an incidental imaging finding. However, it may be complicated by pathologic fracture and rarely by malignant change. It can also be associated with aneurysmal bone cysts (ABCs) [6, 8–12]. ABC is a rare, benign vascular lesion and considered secondary to certain pathological bone lesions. Although the mechanism of the occurrence of FD with ABC is unknown, there are reports that a secondary form of ABC may arise from a disruption in the osseous circulation caused by primary lesion. The development of ABC in FD may hasten the course of presentation and may lead to rapid growth, suggesting a malignant change [5, 10]. We report a case of monostotic FD with ABC changes in a 70-year-old lady who presented with sudden increase in size of the swelling of 20-year duration on the chest wall since 8 months.

## 2. Case Report

**Figure 1:**
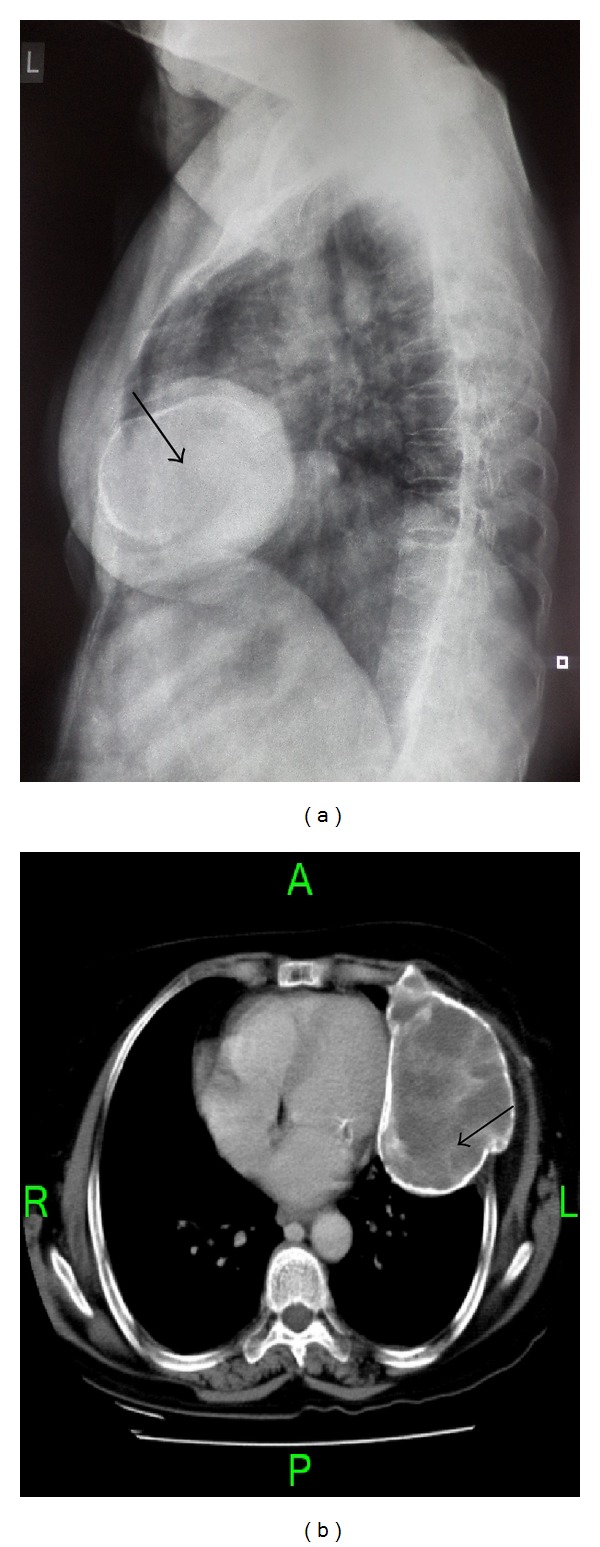
(a) X-ray and (b) CT scan-lobulated, expansile, intramedullary lesion (arrow) with ground-glass centre and thinning of the cortex arising from the anterolateral aspect of the left 5th rib.

**Figure 2:**
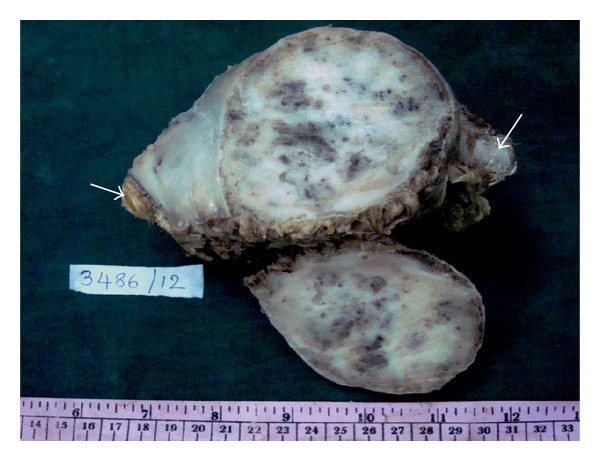
Gross: firm, well defined solid grey white expansile mass replacing the medullary cavity of the rib (arrow) with areas of hemorrhage.

**Figure 3:**
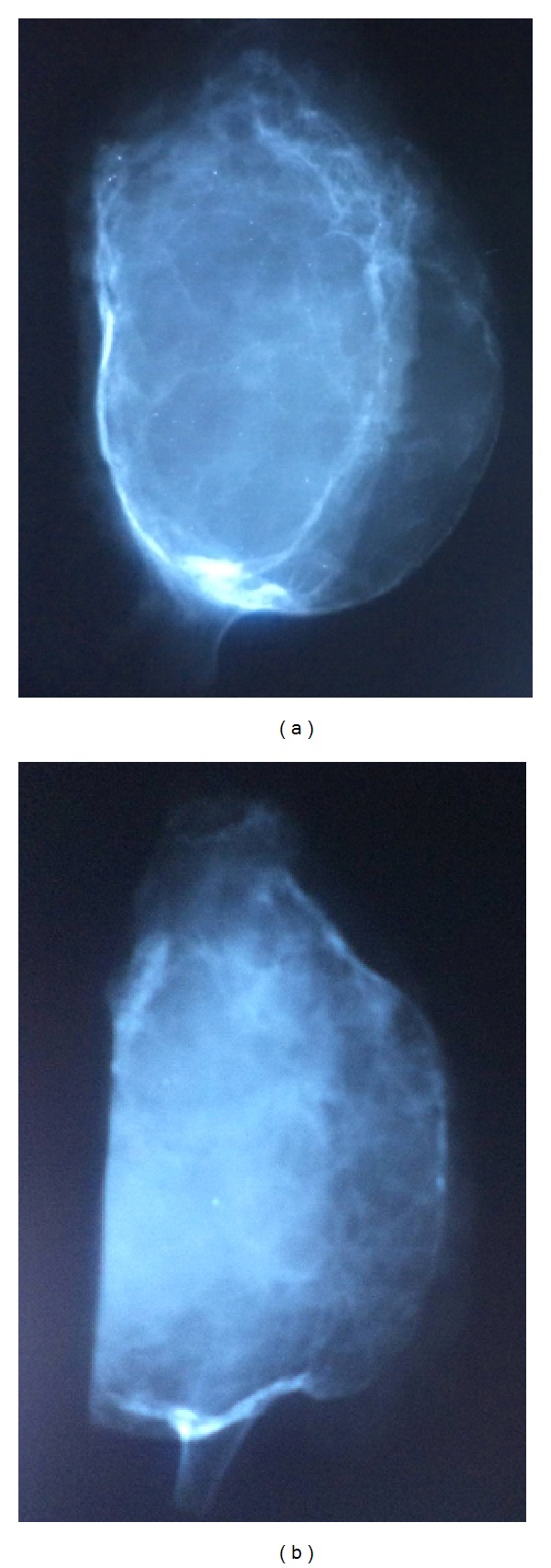
The specimen X-ray ((a) and (b)) displaying the intact lesion with no soft tissue extension.

**Figure 4:**
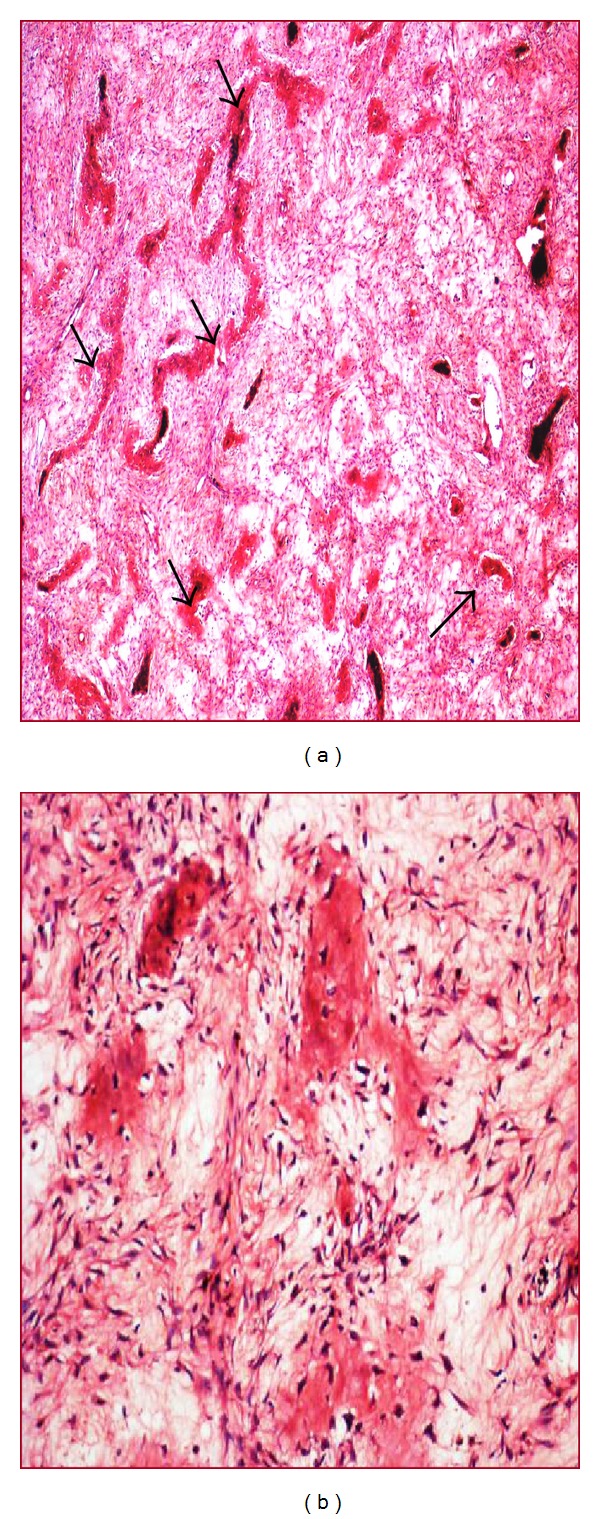
Microscopy, (a) “Chinese alphabet” spicules of woven bone (arrows) separated by abundant fibrous stroma (H&E, 20x). (b) No atypia of stromal cells (H&E, 100x).

**Figure 5:**
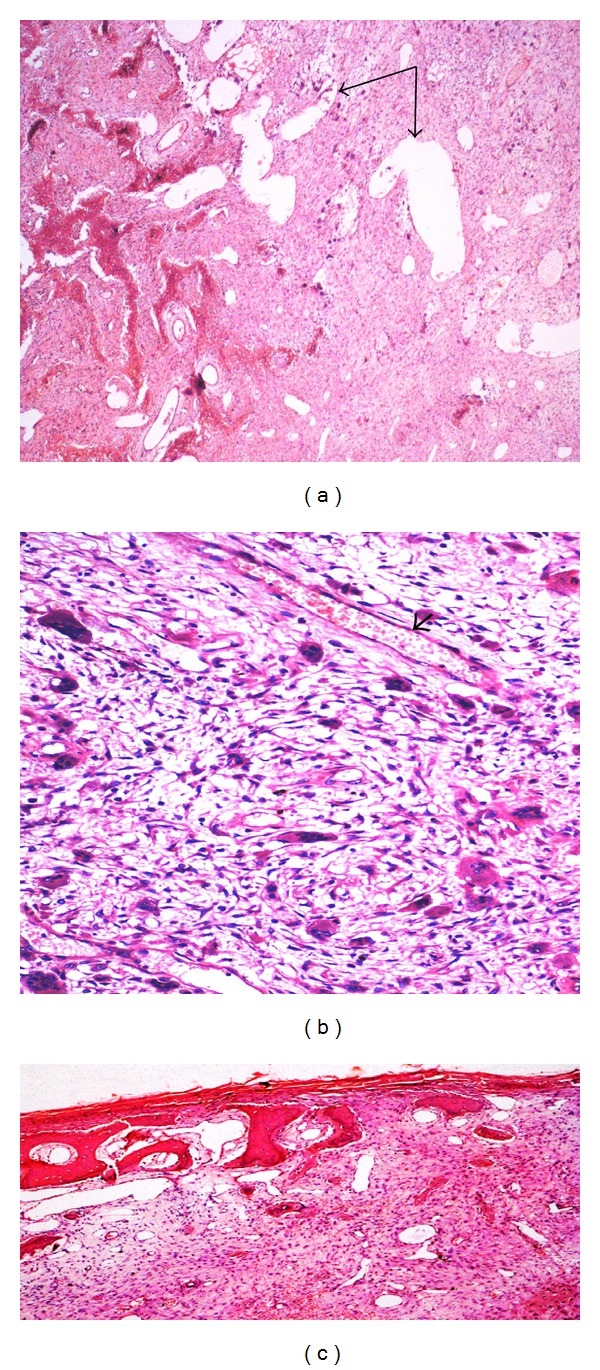
Microscopy, (a) FD (left) With ABC (right) changes—hemorrhagic spaces lined by osteoclast-like multinucleated giant cells (arrows) (H&E, 20x). (b) Osteoclast-like multinucleated giant cells lining hemorrhagic spaces (arrows) (H&E, 100x). (c) Intact periosteum with host lamellar bone and no soft tissue extension (H&E, 20x).

A 70-year-old lady came with sudden increase in size of the swelling of 20-year duration on the left side of chest since 8 months. Radiologically, X-ray and computed tomography (CT) scan showed lobulated, expansile intramedullary lesion with a ground-glass centre and thinning of the cortex arising from the anterolateral aspect of left the 5th rib (Figures 1(a) and 1(b)). The lesion with partial resection of the 5th rib was performed. The gross appearance was a firm, well-defined solid, grey-white expansile mass measuring 10 × 9 cm, and replacing the medullary cavity with areas of hemorrhage. The mass was surrounded by thin bony cortex that has not invaded the surrounding structures (Figure 2). The specimen X-ray confirmed intact of the lesion and no soft tissue extension (Figures 3(a) and 3(b)). Histologically, the lesion consists of osseous and fibrous components. The osseous component consists of disorganized irregular “Chinese alphabet” spicules of woven bone separated by abundant fibrous stroma (Figure 4(a)). The fibrous component is composed of cytologically bland spindle cells with no atypia of stromal cells (Figure 4(b)). Hemorrhagic spaces lined by osteoclast-like multinucleated giant cells of ABC changes were seen (Figures 5(a) and 5(b)). Intact periosteum with host lamellar bone and no soft tissue extension was noted (Figure 5(c)). The final diagnosis was fibrous dysplasia with aneurysmal bone cyst changes.

## 3. Discussion

Fibrous dysplasia (FD) is a benign skeletal disorder, described by Lichtenstein in 1938 and Lichtenstein and Jaffe in 1942. It accounts for 0.8% of primary and 7% of benign bone tumors. FD is monostotic form in 70–80% of cases and polyostotic in 20–30% of cases. Wide age range presentation between 10 and 70 years, with 75% of patients presenting before the age of 30 years [1, 3–5, 8, 9, 11, 12]. It was recognized that both monostotic and polyostotic FD are nonneoplastic processes associated with postzygotic-activating mutations of signal-transducing G proteins encoded by GNAS1 on chromosome 20. Osteoblasts carrying this mutation show increased proliferation and inappropriate differentiation which resulted in fibrotic bone matrix [2, 4, 7, 9, 13]. In its polyostotic form, FD may be associated with McCune-Albright syndrome (polyostotic FD, café-au-lait spots, and endocrine dysfunction) and Mazabraud's syndrome (polyostotic FD and soft-tissue myxomas) [2, 5, 14]. The more extensive and aggressive lesions are commonly found in polyostotic FD, which can affect as few as two bones to as much as 75% of the skeleton, predominantly involving the femur, tibia, pelvis, and foot. Polyostotic lesions progress in number and size until skeletal maturity and then usually become quiescent [4, 8]. Uncomplicated monostotic FD are generally asymptomatic and usually do not cause significant deformity. As a rule, monostotic does not transform to the polyostotic form. Lesions do not increase in size over the time and the disease becomes inactive at puberty [6, 14]. The most common sites of involvement include the ribs (28%), proximal femur (23%), and craniofacial bones (20%). Solitary involvement of other bone is unusual [2, 4, 7]. Monostotic FD in the long bones occurs most frequently in adolescence. In the jaws it is found mainly in early adult life. It presents later in the ribs, probably because it is often asymptomatic in this site [1, 15].

The gross appearance of FD is a firm solid grey-white mass replacing the medullary cavity and surrounded by cortical bone. Histopathologically, the lesion appears well circumscribed and sharply delineated by the host lamellar bone. It is composed of uniformly cellular fibrous tissue containing a proliferation of bland and uniform spindle cells with sparse mitotic activity. Scattered across the fibrous matrix are lamellae or rounded nests of woven bone without significant osteoblastic rimming. There is some morphologic variability in the woven bone spicules. The classic, most commonly seen pattern is that of curvilinear, “Chinese alphabet” spicules of woven bone separated by abundant fibrous stroma. Less commonly, the woven bone may be deposited either in sclerotic, interconnected lamellae, cementoid bodies, or in orderly and parallel spicules [4, 6, 8, 9, 14].

The imaging features of FD are characteristic, although not specific, and depend on the underlying histopathology of a given lesion. Radiographs show unilateral fusiform enlargement of medulla, deformity with cortical thickening, and increased trabeculation. A characteristic “ground-glass” appearance is created by the mixture of woven bone and fibrous components that replace the medullary space. The degree of haziness directly correlates with its underlying histopathology. More radiolucent lesions are composed of predominantly fibrous elements, whereas more radiopaque lesions contain a greater proportion of woven bone [2, 7]. Amorphous or irregular calcification is often seen in the lesion on CT scans. Magnetic resonance (MR) imaging is useful in accurately defining the full extent of the lesion. The signal intensity varies from low to high on T2-weighted images but typically is low in areas of lesion involvement on T1-weighted images [6, 14].

Sudden increase in development of already existing FD will be either due to superimposed ABC or malignant transformation [6, 10, 12, 14]. ABC is an unusual benign mass that has the potential for rapid growth, bone destruction, and extension into adjacent soft tissue. The masses contain a network of multiple blood-filled cysts lined by fibroblasts and multinucleated giant cells of the osteoclast type. Most reported cases are classified as primary, but approximately 20% to 30% are secondary to an identifiable, preexisting lesion. Secondary lesions commonly associated with ABC are giant cell tumor of bone, chondroblastoma, chondromyxoid fibroma, and FD. The haemodynamic changes take place in these preexisting lesions contributes to the formation of arteriovenous fistulae; bone expansion latter follows from the raised intraosseousvascular pressure [5, 10, 12].

Malignant transformation with rapid expansion of the bone has been reported in about 0.5% of patients with monostotic FD but in nearly 4% of those with McCune-Albright syndrome. It may develop after irradiation of the involved bones. Malignant transformation is most common to osteosarcoma, although fibrosarcoma, chondrosarcoma, or malignant fibrous histiocytoma noted [2, 4, 6, 7, 9, 11, 14]. The important differential diagnosis to be considered clinically, radiologically, and histologically is low-grade osteosarcoma. Correlation of the imaging and histology studies is recommended. Radiographic changes that suggest malignancy include lytic regions in previously mineralized zones, intralesional calcification, periosteal reaction, cortical disruption, and a soft-tissue mass [6, 9, 14]. Histologically, low-grade osteosarcoma is more cellular; cytologically, it is more atypical; and mitotically, it is more active than FD. Moreover, the regularly spaced spicules of woven bone seen in FD are not present in osteosarcoma, where malignant osteoid is often deposited in broader and irregular trabeculae [9]. 

Treatment of FD for asymptomatic and stable lesions should be simply monitored. Surgery is indicated only for confirmatory biopsy, correction of deformity, failure of nonsurgical therapy, prevention of pathologic changes, and/or eradication of symptomatic lesions. When surgery is not possible and in the polyostotic form, bisphosphonate therapy is indicated with positive effects exerted on bony density and the reduction of pain. However, a surgical management is preferred for FD of the rib location as simple surveillance can raise the difficult problem of differential diagnosis with malignant tumors [3, 4, 7, 13].

Our case thus gave rise to sudden increase in development of an already existing FD with altered hemodynamic changes by the ABC, clinically presented as malignant lesion. 

## 4. Conclusion

The development of ABC in FD will hasten the course of clinical presentation. FD with ABC should be taken into account in differential diagnosis of the rapidly growing solitary rib lesion. In symptomatic monostotic FD of ribs, the involved segment of bone should beexcised to rule out malignancy and for relief from symptoms. Knowledge of the various appearances, complications, and associations of FD is important to ensure the accurate diagnosis and appropriate management in ribs location.
